# Supplementary material for: Single-molecule force spectroscopy reveals the dynamic strength of the hair-cell tip-link connection
Source: Nat Commun. 2021 Feb 8;12:849. doi: 10.1038/s41467-021-21033-6 (PMC7870652; doi:10.1038/s41467-021-21033-6)
Supplement: Supplementary file 1 — Supplementary Information [file 41467_2021_21033_MOESM1_ESM.pdf]

Supplementary Figures

Supplementary Fig. 1 Force Spectroscopy Material Preparation and Data Analysis

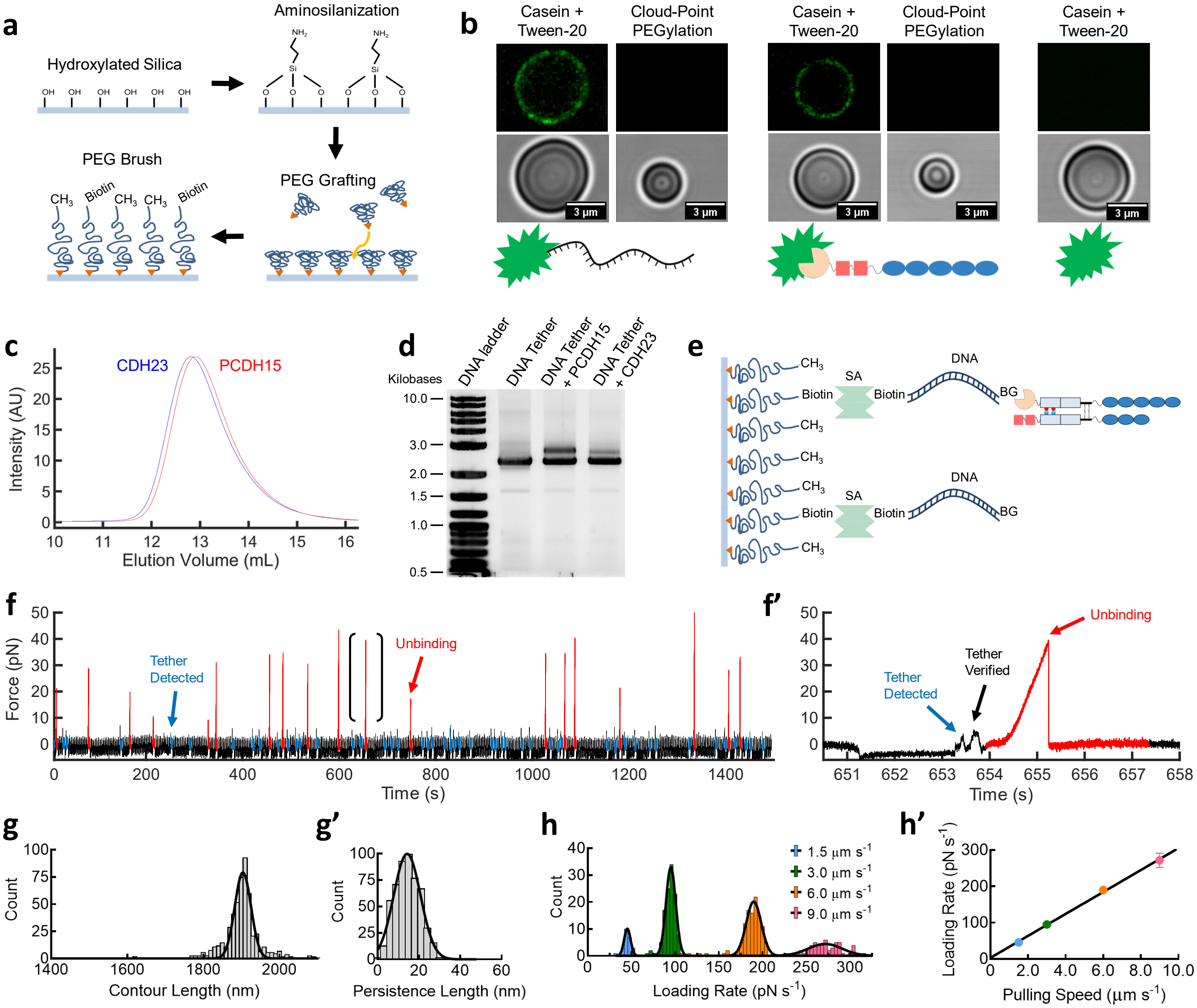

a, Schematic of the cloud-point PEG grafting process. The silica surface of a bead was hydroxylated with a high pH solution and aminosilanized with aminopropylsilane. Amine-reactive NHS-PEG5k-Biotin and NHS-PEG5k-Me were grafted onto the surface in a poor solvent so that the hydrodynamic radius of the PEG molecule was as small as possible. Upon washing the surface with a low-salt saline and detergent solution, the PEG molecules became completely solubilized and formed a PEG brush.

b, Passivation efficacy assessed using fluorescence microscopy. Single stranded DNA and PCDH15 EC1-5 His SNAP fusion proteins were labeled with a single FITC fluorophore and allowed to interact with either plain beads in a non-covalent blocking solution of casein and Tween-20 or with cloud-point PEGylated beads. After washing, the beads were imaged using a confocal microscope at an image plane through the center of the bead. Images were acquired using Olympus FluoView software and analyzed using ImageJ. The experiment was independently repeated twice for biotin-

PEG:mPEG ratios of 1:1 – 1:640 (Methods). Cloud-point PEGylation resulted in no discernable non-specific binding.
c, Size-exclusion chromatography of single-bond tip-link fusion proteins shows monodisperse protein. 100 µL of His-tag purified protein was run on a Superose 6 Increase column at 0.5 mL/min. The fluorescence detector was set to λEX = 280 nm, λEM = 340 nm to detect tryptophan fluorescence.

d, Coupling of purified single-bond fusion proteins to double-stranded 2.385 kb DNA handles. DNA handles containing alternative 5′ dual-biotin and 5′ benzylguanine were reacted with SNAP-tagged fusion proteins, and 100 ng of DNA or DNA-protein was run on a 1% agarose gel. The gel was stained with Sybr Gold DNA stain and imaged with a fluorescent gel scanner. DNA-protein complexes are revealed as an apparent shift in molecular weight. For each tip-link fusion protein, DNA coupling was performed and verified at least twice.

e, Schematic of the DNA-protein tethers immobilized on the PEGylated surface.

f, Force-time series of an entire recording session using single-bond proteins. Peaks highlighted in red represent single-bond unbinding events, and events highlighted in blue represent tethers that were verified by a second pull but unbound before application of the force-loading protocol. **f'**, A representative single-molecule unbinding event, denoted by the brackets in (**f**). Using an automated protocol, beads were brought together for 2 seconds to facilitate bond formation and then pulled apart to detect a tether. Once a tether was detected, the tether length was quickly verified by relaxing and re-applying force, before pulling the tether at a constant velocity of 3000 nm s^{-1} for 3.5 seconds.

g, Histogram of DNA-protein contour lengths obtained from an extensible worm-like chain (WLC) fit of force-extension data for single-bond measurements above 22.7 pN s^{-1}. Events are plotted in 10-nm bins and fit with a Gaussian curve (1904 ± 23 nm, mean ± SD). Contour lengths form a single distribution around the predicted contour length of 1912 nm, indicating the measurement of individual tethers containing the correct components. **g'** Histogram of DNA-protein persistence lengths calculated from the same WLC fits, plotted in 3-nm bins and fit with a Gaussian curve (14.3 ± 6.5 nm, mean ± SD). The persistence length of a polymer is a metric of its bending stiffness and can be used to check for the presence of multiple tethers during an experiment.

h, Histograms of loading rates at different pulling speeds obtained from an extensible WLC fit of force-extension data for single-bond measurements. Each histogram is plotted in 5 pN s^{-1} bins and each was fit with a Gaussian curve (1.5 µm s^{-1}: 45.4 ± 3.7 pN s^{-1}, 3.0 µm s^{-1}: 95.1 ± 5.6 pN s^{-1}, 6.0 µm s^{-1}: 189.8 ± 8.6 pN s^{-1}, 9.0 µm s^{-1}: 271.5 ± 19.5 pN s^{-1}, mean ± SD). **h'** Fit loading rate from (**h**) plotted against pulling speed. A linear regression fit well to the data (Slope = 0.03, Y-intercept = 3.229, R^2 = 0.9983).

Supplementary Fig. 2 Methods for Biolayer Interferometry

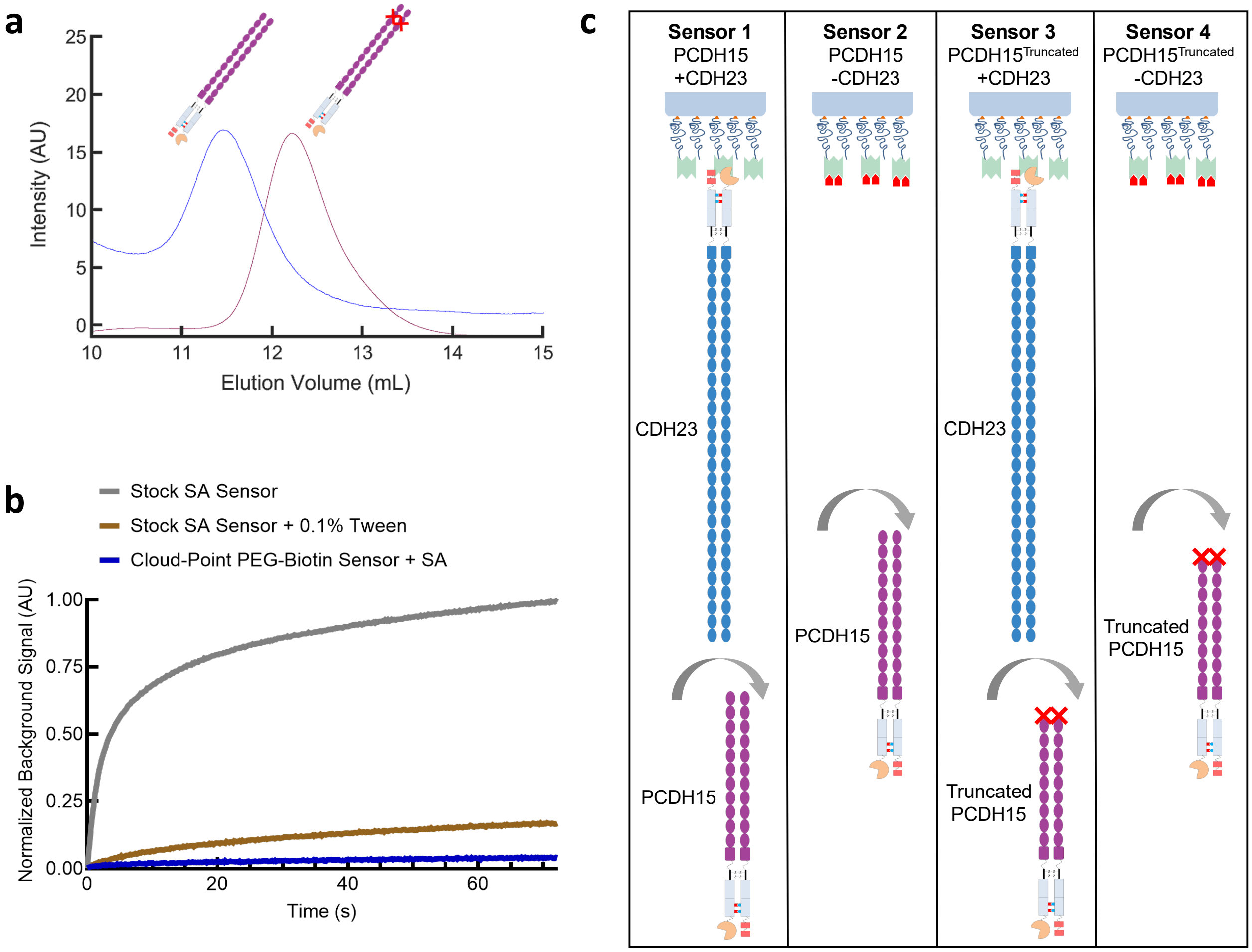

a, Size-exclusion chromatography of full-length double stranded PCDH15 (blue trace) and the PCDH15 control proteins lacking the EC1-2 binding domains (red trace). Proteins were labeled through their SNAP tags with BG-Alexa 647 and the fluorescence detected with 650 nm excitation and 665 nm emission. Control proteins have a lower apparent molecular weight than WT proteins and each protein forms a single peak.

b, The passivation efficacy of cloud-point PEGylated BLI sensors. Stock streptavidin BLI sensors (ForteBio) in TBS-Tween+Ca^{2+} buffer (Methods), with or without 0.1% Tween-20, were compared for passivation efficiency against Cloud-Point PEG-Biotin sensors coated with streptavidin. Each sensor was dipped into 100 nM PCDH15 protein and the signal resulting from background bindings was measured for 75 seconds.

c, Biolayer interferometry signal subtraction strategy. Biosensors were coated with PEG-streptavidin and loaded with full-length double-stranded CDH23 (sensors 1&3). The drift-control biosensors were biocytin-quenched and not CDH23-loaded (sensors 2&4). The drift-subtracted truncated control signal (sensor 3 – sensor 4) was then subtracted from drift-subtracted full-length experimental signal (sensor 1 – sensor 2)

Supplementary Fig. 3 Alternative Lifetime Estimates and Modeling of Force-Dependent Concentration

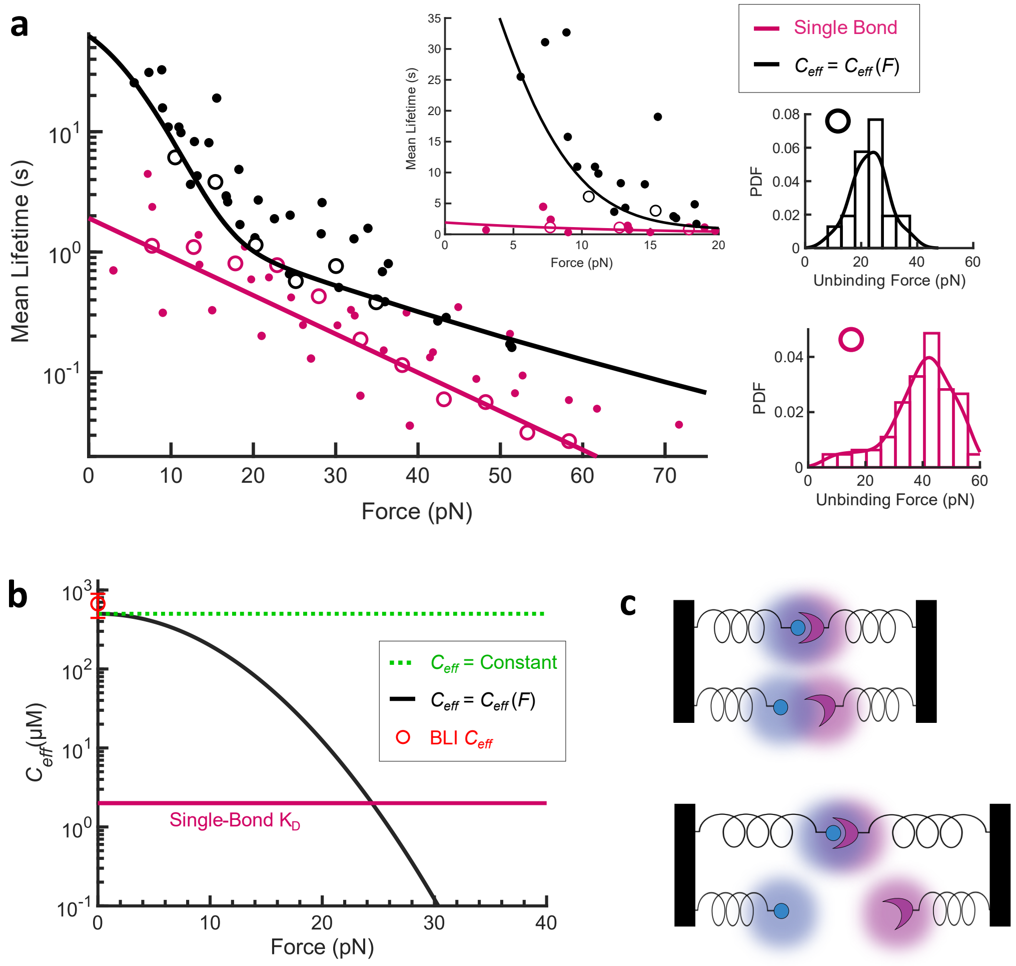

a, Tip-link lifetimes calculated directly from unbinding force data are consistent with lifetimes calculated from fits by the models. *inset*, plotting with the y-axis on a linear scale highlights the difference in calculated lifetimes over the rebinding domain for full-length dimers. right, sample histograms of systematically binned unbinding forces for single bonds (magenta) and double bonds (black) overlaid with a kernel smooth density function. To estimate the apparent off-rate as a function of force, $k_{off}(F_i)$, we used the following equation: $\frac{1}{k_{off}(F_i)} = \frac{\Delta F}{l_r}\frac{\sum_{j=i}^{N} n_j}{n_i}$, where F_i is the ith bin center, l_r is the loading rate, and n_i is the number of counts in bin i [49,50]. The estimated mean lifetime as a function of force $\frac{1}{k_{off}(F_i)}$ for each bin is plotted on the calculated mean lifetime plot from Fig. 3a.

b, Effective concentration of the unbound binding domains as a function of force calculated from the model fit. C_{eff} decreases with a Gaussian decay dependent on the compliance parameter f_c. At ~25 pN, C_{eff} becomes less than the concentration of the equilibrium dissociation constant for a single bond[11,26,46]. The extrapolated C_{eff} at zero force matches well with the biolayer interferometry measurements (red circle).

c, A schematic of the force-dependent concentration model (Methods). The local concentration depends on the elasticity of the tip link filaments. When one bond is broken, the separation between unbound domains is further increased as the force is transmitted through the bound strand.

Supplementary Fig. 4 Rupture Force Histograms and Probability Density Estimates

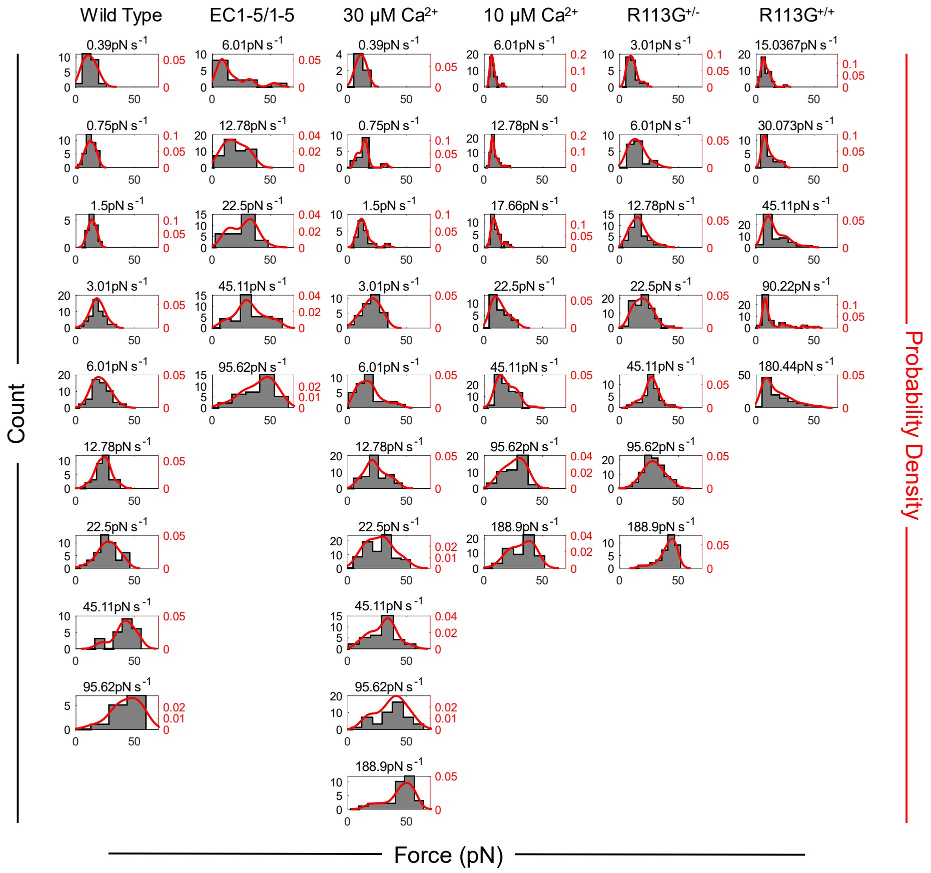

Histograms of rupture forces for all dimeric unbinding data at each tested loading rate. Unbinding forces at each loading rate were systematically binned using the Freedman–Diaconis rule to yield N total bins of width ΔF. Bin centers were systematically chosen to yield the most likely unbinding force with the maximum number of counts. A kernel smoothing function was used to estimate the unbinding probability density as a function of force; the result is overlaid on the binned unbinding force data in red[89-91].

Supplementary Table 1 Fit Parameters from Force Spectroscopy Models

Condition	Mean Lifetime (s)	Single-bond f_β (pN)	Single-bond k_{off} (s^{-1})	Single-bond k_{on} (M^{-1} s^{-1}) x 10^4	C_{eff} (mM)	f_c (pN)	Number of Unbinding Events
EC1-5/3-5 Single Bond 2 mM Ca2+	1.9 ± 0.4	13.5 ± 2.0	0.5 ± 0.1	--	--	--	n = 411
EC1-5/3-5 Single Bond 50 μM Ca^{2+}	0.9 ± 0.1	16.8 ± 0.8	1.2 ± 0.1	--	--	--	n = 174
Full Length Dimer 2 mM Ca2+	62 ± 17	13.5	0.5	7.0	0.5 ± 0.1	10.0 ± 1.8	n = 247
EC1-5/1-5 Dimer 2 mM Ca^{2+}	91 ± 261	13.5	0.5	7.0	0.7 ± 2.0		n = 159
Full Length Dimer 30 μM Ca2+	72 ± 24	11.4 ± 3	0.5 ± 0.1	7.0	0.6 ± 0.1	10 ± 4	n = 339
Full Length Dimer 10 μM Ca2+	6 ± 5	22.0 ± 15.7	2.6 ± 0.8	7.0	0.9 ± 0.7	2.9 ± 1.1	n = 344
Full Length Dimer PCDH15 R113G +/-	40 ± 17	1.8 ± 0.3	1.4 ± 0.6	10 ± 4	0.5	10.5 ± 3.0	n = 316
Full Length Dimer PCDH15 R113G +/+	24 ± 10	1.8 ± 0.3	1.4 ± 0.6	10 ± 4	0.5	10.5 ± 3.0	n = 264

Kinetic fit parameters of force spectroscopy data (mean ± SD). Values in red are held constant in the fit. R113G+/+ and R113G+/- data were simultaneously fit.

Supplementary Table 2 PCR Primers

Name	Sequence (5'-3')
F DNA tether	AACATCCAATAAATCATACAGGCAAG
R DNA tether	TTCTGCTGGTGGTTCGTTCG
F Pcdh15	ATGTTCCTACAGTTTGCTGTC
F Pcdh15 EC3-5	GGAGATGACCTGGGACCTATG
R Pcdh15 EC3-5	CTGGCCCCAGCTGACTACCAAGAG
R Pcdh15 EC1-5	CTGGTTGTTAGGAGGAAGCACC
R Pcdh15 Full Extracellular Domain	TTCTGTGTACCCCAAGC
F Pcdh15 R113G Mutagenesis	TCATGAAGTACGCATCGTGGTG
R Pcdh15 R113G Mutagenesis	CACCACGATGCGTACTTCATGA
F Cdh23	ATGAGGTACTCCCTGGTCAC
F Cdh23 EC3-5	ATGCAAGACATGGATCCTATC
R Cdh23 EC3-5	CTGGCCCCAGGATCCAG
R Cdh23 EC1-5	ATCCAGCACATTGATCCGG
R CDh23 Full Extracellular Domain	GGCAGACATGTCATCCGGC
F hIgG Fc1	GACAAAACTCACACATGCCCAC
R hIgG Fc1	TTTACCCGGAGACAGGGAGAG
F SNAPtag	ATGGACAAAGACTGCGAAATG
R SNAPtag	ACCCAGCCCAGGCTTGC
