# Supplementary material for: Single-molecule force spectroscopy reveals the dynamic strength of the hair-cell tip-link connection
Source: Nat Commun. 2021 Feb 8;12:849. doi: 10.1038/s41467-021-21033-6 (PMC7870652; doi:10.1038/s41467-021-21033-6)
Supplement: Supplementary file 4 — Reporting Summary [file 41467_2021_21033_MOESM4_ESM.pdf]

nature research

Corresponding author(s): David P. Corey
Wesley P. Wong

Last updated by author(s): Dec 15, 2020

Reporting Summary

Nature Research wishes to improve the reproducibility of the work that we publish. This form provides structure for consistency and transparency in reporting. For further information on Nature Research policies, seeAuthors & Referees and theEditorial Policy Checklist .

Statistics

For all statistical analyses, confirm that the following items are present in the figure legend, table legend, main text, or Methods section.

n/a	Confirmed	
☐	☒	The exact sample size (*n*) for each experimental group/condition, given as a discrete number and unit of measurement
☐	☒	A statement on whether measurements were taken from distinct samples or whether the same sample was measured repeatedly
☐	☒	The statistical test(s) used AND whether they are one- or two-sided<br>*Only common tests should be described solely by name; describe more complex techniques in the Methods section.*
☒	☐	A description of all covariates tested
☒	☐	A description of any assumptions or corrections, such as tests of normality and adjustment for multiple comparisons
☐	☒	A full description of the statistical parameters including central tendency (e.g. means) or other basic estimates (e.g. regression coefficient) AND variation (e.g. standard deviation) or associated estimates of uncertainty (e.g. confidence intervals)
☐	☒	For null hypothesis testing, the test statistic (e.g. *F*, *t*, *r*) with confidence intervals, effect sizes, degrees of freedom and *P* value noted<br>*Give P values as exact values whenever suitable.*
☒	☐	For Bayesian analysis, information on the choice of priors and Markov chain Monte Carlo settings
☒	☐	For hierarchical and complex designs, identification of the appropriate level for tests and full reporting of outcomes
☒	☐	Estimates of effect sizes (e.g. Cohen's *d*, Pearson's *r*), indicating how they were calculated

Our web collection on statistics for biologists contains articles on many of the points above.

Software and code

Policy information about availability of computer code

Data collection	Custom software written in LabVIEW(Version 2018) was used for optical tweezer data collection. Monte Carlo simulations were performed with custom software in MATLAB (Version 2018b). Biolayer interferometery measurements were acquired using ForteBio Octet Data Acquisition Software (Version 10.0). Confocal images were acquired using Olympus FluoView software (Version 4.2).
Data analysis	Custom software written in MATLAB (Version 2018b) was used for optical tweezer data analysis and modeling, and is available from GitHub [https://github.com/Corey-Lab/Mulhall-et-al-Nature-Communications]. Biolayer interferometery measurements were analyzed using ForteBio Octet Data Analysis Software (Version 10.0) and Graphpad Prism (Version 8.1). Confocal and gel images were analyzed using ImageJ (Version 1.52n). DNA sequences were analyzed using SnapGene (Version 5.1).

For manuscripts utilizing custom algorithms or software that are central to the research but not yet described in published literature, software must be made available to editors/reviewers. We strongly encourage code deposition in a community repository (e.g. GitHub). See the Nature Research guidelines for submitting code & software for further information.

Data

Policy information about availability of data

All manuscripts must include a data availability statement. This statement should provide the following information, where applicable:

- Accession codes, unique identifiers, or web links for publicly available datasets
- A list of figures that have associated raw data
- A description of any restrictions on data availability

Methods are as described in the text. Most data are available as supplementary materials. The datasets generated during and/or analysed during the current study are available from the corresponding author on reasonable request.

Field-specific reporting

Please select the one below that is the best fit for your research. If you are not sure, read the appropriate sections before making your selection.

☒ Life sciences ☐ Behavioural & social sciences ☐ Ecological, evolutionary & environmental sciences

For a reference copy of the document with all sections, see nature.com/documents/nr-reporting-summary-flat.pdf

Life sciences study design

All studies must disclose on these points even when the disclosure is negative.

Sample size	No sample size calculation was performed. Sample sizes were deemed sufficient based upon the magnitude of the estimation errors for force spectroscopy data.
Data exclusions	For measurements of single tip-link bond interactions, any force-extension curves that displayed DNA overstretching (rare) was excluded as background. No other data was excluded from analysis.
Replication	For most experiments, biological replicates were performed (e.g. a new batch of protein/DNA tether was produced from scratch). For all experiments, experimental replicates were performed (i.e. new samples/recording chambers were prepared). All replication attempts were successful, and replications were separated in time by at least two days.
Randomization	Randomization was not relevant to this study. All samples were subjected to the same experimental procedures.
Blinding	Blinding was not relevant to this study because this study because all experimental data was included for analysis. All data collection procedures were standardized, so no subjective manipulation was possible.

Reporting for specific materials, systems and methods

We require information from authors about some types of materials, experimental systems and methods used in many studies. Here, indicate whether each material, system or method listed is relevant to your study. If you are not sure if a list item applies to your research, read the appropriate section before selecting a response.

Materials & experimental systems

n/a	Involved in the study	
☒	☐	Antibodies
☐	☒	Eukaryotic cell lines
☒	☐	Palaeontology
☒	☐	Animals and other organisms
☒	☐	Human research participants
☒	☐	Clinical data

Methods

n/a	Involved in the study	
☒	☐	ChIP-seq
☒	☐	Flow cytometry
☒	☐	MRI-based neuroimaging

Eukaryotic cell lines

Policy information about cell lines

Cell line source(s)	Thermo Fisher Scientific - Expi293 cells.
Authentication	None of the cell lines were authenticated.
Mycoplasma contamination	Tested negative by the supplier.
Commonly misidentified lines (See ICLAC register)	There are no commonly misidentified cell lines used in this study.
